# Tandem extracorporeal blood purification therapies for sepsis and acute kidney injury in critically ill children: a state-of-the-art review

**DOI:** 10.1080/0886022X.2026.2653939

**Published:** 2026-04-14

**Authors:** Parth Shirode, Sidharth Kumar Sethi, Daniel Nascimento, Shay Tanna, Ethan Johnson, Kareem Safadi, Tara Beck, Sevcan A. Bakkaloglu, Amrit Kirpalani, Patricia Raimer, Michael L. Moritz, Rupesh Raina

**Affiliations:** ^a^ Department of Pediatric Nephrology, Akron Children’s Hospital, Akron, OH, USA; ^b^ Pediatric Nephrology, Medanta The Medicity, Medanta Institute of Kidney and Urology, Gurgaon, Haryana, India; ^c^ Department of Medicine, College of Medicine, Northeast Ohio Medical University, Rootstown, OH, USA; ^d^ Department of Nephrology, Akron Nephrology Associates at Cleveland Clinic Akron General Medical Center, Akron, Ohio; ^e^ Department of Neonatology and Pediatric Nephrology, University of Pittsburgh Medical Center, Pittsburgh, PA, USA; ^f^ Department of Pediatric Nephrology, Gazi University Faculty of Medicine, Ankara, Turkey; ^g^ Division of Pediatric Nephrology, Children’s Hospital, London Health Sciences Centre, London, Ontario, Canada; ^h^ Division of Critical Care Medicine, Department of Pediatrics, Akron Children’s Hospital, Akron, OH, USA

**Keywords:** Pediatric critical care, acute kidney injury, sepsis, extracorporeal blood purification, continuous renal replacement therapy, extracorporeal membrane oxygenation

## Abstract

Tandem extracorporeal blood purification and support therapies integrate established therapies such as continuous renal replacement therapies (CRRT), extracorporeal membrane oxygenation (ECMO), intermittent or prolonged renal replacement therapies with emerging adsorptive, immunomodulatory, and organ-support technologies. These approaches are increasingly applied in critically ill children with complex multi-organ dysfunction, particularly in the settings of severe sepsis, hyperinflammatory syndromes, acute kidney injury, liver failure, and refractory cardiorespiratory compromise. Despite growing clinical adoption, most tandem applications remain off-label in pediatrics, with evidence largely limited to case reports, single-center experiences, and small observational cohorts, and with few standardized protocols to guide practice.This state-of-the-art review synthesizes available pediatric and relevant adult literature to examine the mechanistic rationale, technical integration, clinical indications, and reported outcomes of tandem extracorporeal strategies beyond conventional CRRT–ECMO–therapeutic plasma exchange combinations. We focus on under-reported and emerging modalities, including hemoperfusion and adsorptive hemofilters integrated with CRRT or ECMO, leukapheresis combined with renal replacement or extracorporeal life support, selective cytopheretic and immunomodulatory devices, extracorporeal liver support platforms, and novel miniaturized and translational technologies. Practical considerations related to circuit configuration, anticoagulation strategies, and extracorporeal volume are discussed, alongside safety considerations unique to pediatric populations. While early experiences suggest feasibility and potential benefit, current evidence is insufficient to establish definitive efficacy or survival benefit. This review highlights critical knowledge gaps, implementation barriers, and research priorities, and aims to support the development of standardized, evidence-based guidelines to promote safe, effective, and reproducible use of tandem extracorporeal therapies in pediatric critical care.

**Clinical implications**
1. Careful patient selection is critical: Tandem extracorporeal blood purification/support therapies should be reserved for critically ill children with refractory shock or multi-organ dysfunction after conventional therapies fail, given limited pediatric efficacy data.
2. Timing may influence outcomes: Earlier initiation during the hyperinflammatory phase (rather than rescue use) may improve hemodynamics and organ recovery, but requires validation in prospective pediatric studies.
3. Therapy must be indication-driven: Device choice (e.g., cytokine adsorption vs endotoxin removal vs immunomodulation) should align with the dominant pathophysiology rather than a “one-size-fits-all” approach.
4. Hemodynamic benefits may precede survival benefit: Many tandem therapies show rapid improvement in vasoactive requirements and inflammatory markers, even when mortality benefit remains unproven.
5. Anticoagulation strategies require expertise: Regional citrate or heparin protocols must be individualized to minimize bleeding, clotting, and circuit downtime in complex tandem configurations.
6. Drug clearance and adsorption matter: Adsorptive membranes can significantly remove antibiotics and other critical drugs, mandating therapeutic drug monitoring and proactive dose adjustment.
7. Extracorporeal volume limits use in small children: High priming volumes may necessitate blood priming and restrict application in neonates and infants, reinforcing the need for pediatric-specific devices.
8. Safety appears acceptable but data are limited: Reported pediatric experiences suggest feasibility with few device-related adverse events, though conclusions are constrained by small cohorts and observational designs.
9. Standardized protocols reduce variability: Institution-level guidelines for indications, circuit setup, monitoring, and de-escalation are essential to ensure reproducibility and safety.
10. Multidisciplinary coordination is essential: Successful implementation requires close collaboration among intensivists, nephrologists, perfusionists, pharmacists, and nursing teams to balance benefits, risks, and resource use.

## Introduction

Combinations of extracorporeal purification and support therapies (EBP/ST), such as continuous renal replacement therapy (CRRT), extracorporeal membrane oxygenation (ECMO), therapeutic plasma exchange (TPE), and adsorption techniques, are increasingly utilized as off-label therapies to manage critically ill patients with complex multi-organ failure. Although evidence of EBP/ST in children is limited, the existing pediatric and adult cohort studies report that TPE combined with CRRT or ECMO is feasible and generally safe, with observed survival rates ranging from 50% to 82% depending on patient complexity [1]. Notable applications of these tandem therapies include the management of sepsis and TAMOF, including hyperinflammatory and hypo-inflammatory states, where tailored approaches are used to address heterogeneous clinical presentations [2]. However, concerns include the frequent use of off-label extracorporeal therapies in critically ill children without established evidence, often based on clinician judgment and case-by-case risk-benefit decisions. This underscores the urgent need for standardized guidelines and regulatory oversight [3,4].

Current literature is insufficient, especially for the pediatric population, and additionally, much of the existing literature on prescription strategies is focused on combinations of TPE, CRRT, and ECMO [2,5]. By contrast, published experience with leukapheresis during CRRT and ECMO, hemoperfusion combined with intermittent or prolonged renal replacement therapy, adsorption hemofilters integrated into CRRT circuits, extracorporeal liver support during ECMO, and other EBP/ST modalities is limited to single-center series and isolated case reports [6]. These tandem strategies aim to reduce systemic inflammation, correct metabolic derangements, and deliver organ-specific support using advanced materials and technologies when conventional tandem therapy modalities prove inadequate. This review focuses on these under-reported modalities and proposes research priorities to guide their safe and standardized adoption in critical care practice. The aim is to inform future investigations and encourage the development of evidence-based guidelines that improve care and outcomes in critically ill children.

It is important to distinguish mechanistic plausibility from proven clinical efficacy. While many extracorporeal blood purification strategies demonstrate reductions in circulating cytokines, endotoxins, or other inflammatory mediators, such biomarker clearance does not inherently translate into improved survival, reduced organ failure, or shortened ICU stay. Most available pediatric evidence remains observational, and controlled trials demonstrating definitive outcome benefit are lacking. Therefore, throughout this review, reductions in inflammatory mediators should be interpreted as biologically plausible mechanisms rather than established evidence of improved patient-centered outcomes.

## Continuous renal replacement therapy (CRRT) with hemoperfusion

CRRT is indicated for acute kidney injury (AKI), fluid overload (FO), severe electrolyte imbalance, and toxin removal in cases such as septic shock, uremic complications, metabolic abnormalities, and intoxications [7,8]. Varying modalities of CRRT include continuous veno-venous hemofiltration (CVVH), continuous veno-venous hemodialysis (CVVHD), and continuous veno-venous hemodiafiltration (CVVHDF) [9,10]. Renal support is frequently administered to children with sepsis to mitigate the effects of systemic inflammatory response syndrome (SIRS). In cases of AKI in critically ill COVID-19 patients, CRRT is considered a supplementary approach to eliminate pro-inflammatory agents, aiming to decrease mortality rates [11]. CRRT demonstrates significant effectiveness in managing severe COVID-19 cases by eradicating endotoxins and cytokines, thereby stabilizing the patient’s hemodynamic condition [12]. To supplement the renal replacement in these patients, various blood filters based on hemoperfusion technique are utilized in conjunction with CRRT, and are selected based on specific therapeutic objectives.

Hemoperfusion is an extracorporeal blood purification technique that removes unwanted plasma solutes through direct adsorption onto sorbent materials [13]. Earlier, its use was limited by bio-incompatibility issues, such as thrombocytopenia and leukopenia. Recent advances in sorbent production and surface coating technologies have markedly improved safety and compatibility [13].

## Tandem CRRT with adsorptive filtration for cytokine and endotoxin removal

**Figure 1.**
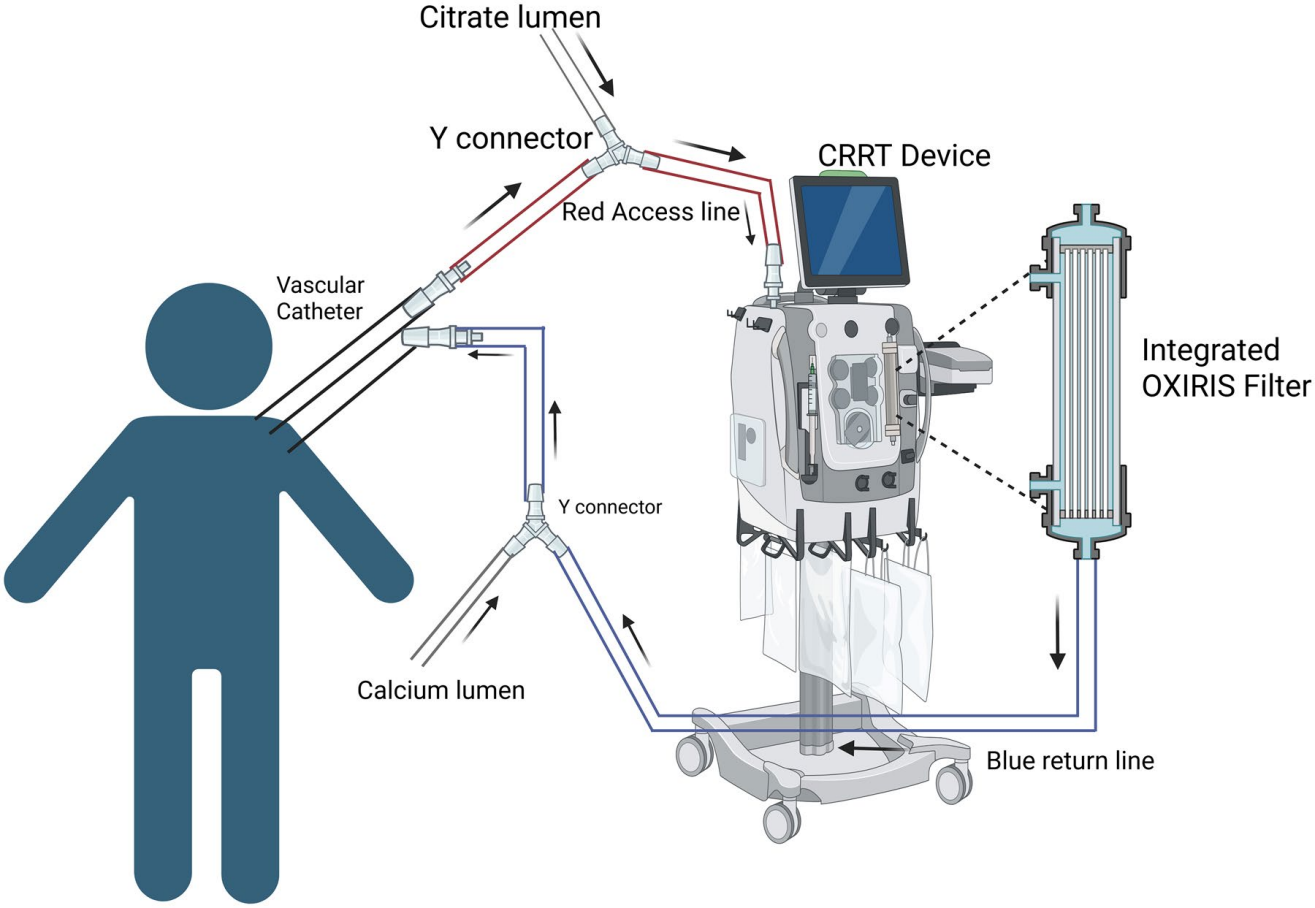
CRRT with the Oxiris filter. Circuit diagram showing integration of the Oxiris filter with CRRT Created in BioRender. Hu, J. (2026) https://BioRender.com/pc45pzs

**Figure 2.**
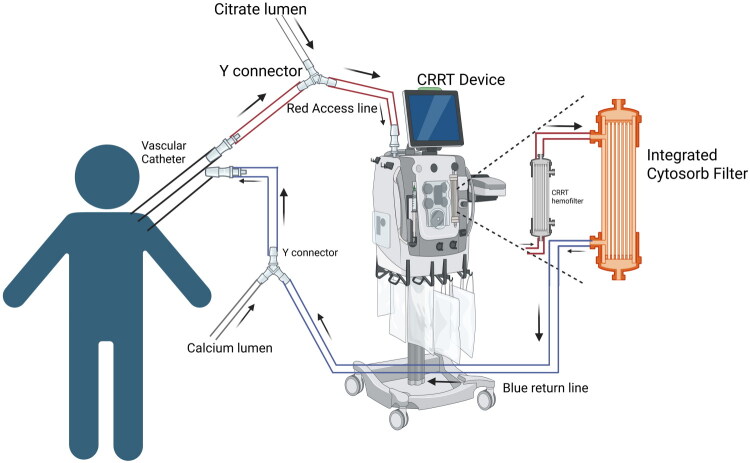
CRRT with the Cytosorb filter. Circuit diagram showing integration of Cytosorb filter with CRRT Created in BioRender. Hu, J. (2026) https://BioRender.com/zijrcpt

For cytokine elimination, various filters such as CytoSorb^®^, HCO/MCO, and HA330 are employed, polymyxin B (PMX) and coupled plasma filtration adsorption (CPFA) are utilized for endotoxin removal, whereas oXiris^®^ is applied for removing both cytokines and endotoxins [14]. This section will address oXiris^®^, CytoSorb^®^, and polymyxin B (PMX) filters, while additional hemofilters will be discussed in subsequent sections. oXiris^®^ is a filtration apparatus designed for eradicating both cytokines and endotoxins through membrane ionic interactions [12,14]. It is made of an acrylonitrile and sodium methallyl sulfonate copolymer (AN69 copolymer). It is covered with polyethyleneimine and heparin, the latter at an average density of 4500 ± 1500 IU/m2 [12,14]. Each layer of the oXiris contributes specifically to its filtration function: the innermost layer comprises a negatively charged AN69 copolymer, followed by a positively charged polyethyleneimine layer, and finally a layer of heparin [12] **(**Figure 1**)**. In contrast, CytoSorb^®^, a filtration device composed of Polystyrene divinylbenzene copolymer microporous beads (coated with polyvinylpyrrolidone) [14], eliminates cytokines and other inflammatory mediators through hydrophobic interactions. It is exclusively applied in a veno-venous configuration within a blood pump circuit [12,14], **(**Figure 2**).**

Both filtration devices are FDA-approved only for adult patients 18 years of age or older. *Morin* et al. *(2023)* reported that hemofiltration and extracorporeal blood purification using oXiris^®^ can be effective in pediatric patients with vasodilatory shock, leading to rapid hemodynamic improvement in selected cases [15]. Raina et al. summarized the related studies presenting positive outcomes of combining Cytosorb^®^ with CRRT within pediatric populations [14]. Additionally, recent case reports propose potential benefits of combining blood filtration devices, such as oXiris^®^ and CytoSorb^®^, with CRRT in pediatric patients. For example, Bottari et al. showed hemoperfusion with CytoSorb, combined with CRRT, significantly reduced IL-6 and IL-10 in critically ill children with septic shock, with no cases of chronic kidney disease or CRRT requirement at 30 days post-discharge [16]. These findings are summarized in Table 1, illustrating the potential emerging role of filtration devices in this population. However, these reports are limited to small observational cohorts and case reports, and no controlled pediatric trials have demonstrated a survival or long-term organ recovery benefit attributable specifically to cytokine adsorption.

**Table 1.** Case reports describing efficiency of blood purification devices in tandem with high flow CRRT in children.

*Case Report*	*Details*	*Treatment Modality*	*Outcome*
Ying et al. (2023) 17	6-year-old boy with septic shock	oXiris^®^ hemofilter in CRRT	Tandem therapy significantly reduced inflammatory biomarkers, and vasopressor dependency, and improved kidney function.
Phan et al. (2022) 18	9-year-old boy with recurrent COVID-19-induced fulminant myocarditis	oXiris^®^ hemofilter in CVVH alongside VA-ECMO	His left ventricular ejection fraction (LVEF) improved and the patient made a full recovery without sequelae.
Lalwani et al.(2022) 19	7-year-old child with MIS-C	oXiris^®^ membrane in CRRT with cytokine filter	Child was discharged within a week, with decreased coronary dilation, improved cardiac function, and resolved pericardial effusion.
Hui et al. (2022) 20	14-year-old male with MIS-C and rhabdomyolysis-associated acute kidney injury	Cytosorb^®^ hemoadsorption column followed by oXiris in CKRT	Significant reductions in inflammatory markers (IL-6, IL-8, TNF-α), improved cardiac function, and stabilization of the patient’s clinical condition.

While these reports highlight the potential benefits of adsorptive filtration devices in pediatrics, providers should be aware of the device priming volume (Oxiris, 193 mL; Cytosorb, 150 mL), which may result in a large extracorporeal therapy volume and limit use in smaller children, or which may necessitate additional blood priming. Extracorporeal volume (ECV) represents a critical safety consideration in pediatric tandem therapies. Estimated circulating blood volume (CBV) in infants is approximately 70–80 mL/kg [21,22]; thus, in a 5 kg infant (CBV ≈ 350–400 mL), a 150 mL CytoSorb^®^ cartridge [23] constitutes approximately 37–43% of CBV, and an oXiris^®^ filter (≈193 mL priming volume) [24] may approach 48–55% of CBV if not blood-primed. Even in a 10 kg child (CBV ≈ 700–800 mL), these devices may account for 19–28% of total blood volume. When combined with CRRT or ECMO circuits, cumulative ECV can surpass 20–30% of circulating volume, increasing the risk of hemodilution, hypotension during circuit initiation, transfusion exposure, and reduced circuit tolerance in hemodynamically unstable patients. These risks are amplified in neonates and small infants and may necessitate blood priming, staged circuit initiation, or device selection adjustments.

In addition to cytokine-directed cartridges mentioned above, endotoxin-targeted adsorptive devices have also been explored in tandem with CRRT. The polymyxin B (PMX) filter, can be incorporated into CRRT and ECMO circuits to help remove inflammatory mediators and neutralize endotoxins from circulation, which can help attenuate septic shock progression [25]. In a case series of children with refractory septic shock, by *Saetang* et al. the Polymyxin B-hemoperfusion (PMX-HP) filter demonstrated decreases in PELOD-2 and vasoactive-inotropic scores, as well as serum lactate concentrations, within 72 h of initiation [26]. There has been no evidence in the literature on the use of tandem CRRT with PMX-HP filter in children. However, such a tandem approach could provide a dual-modality approach—mechanical renal support with targeted endotoxin adsorption. Thus, endotoxin removal in children should be interpreted as a mechanistic strategy to modulate septic physiology rather than as a proven survival-enhancing therapy. While technically feasible and safe, definitive survival advantages remain unproven, emphasizing the need for better patient selection in ongoing trials.

**Figure 3.**
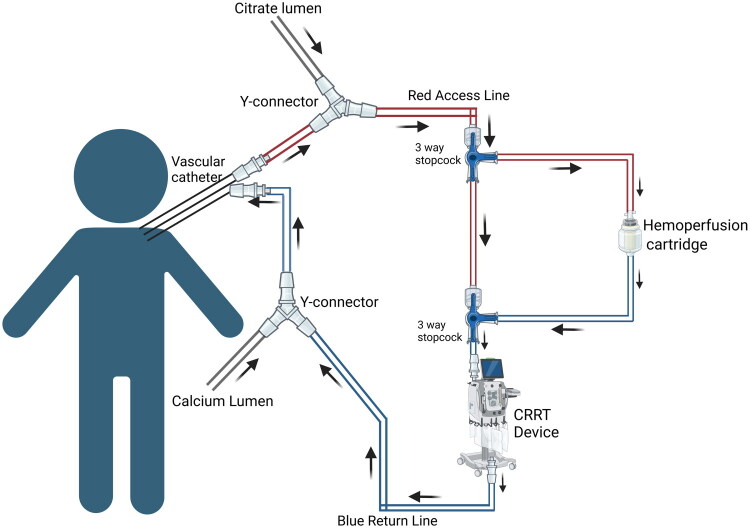
Hemoperfusion with CRRT. Circuit diagram showing integration of Hemadsorption with CRRT Created in BioRender. Hu, J. (2026) https://BioRender.com/ngrvx7b

**Figure 4.**
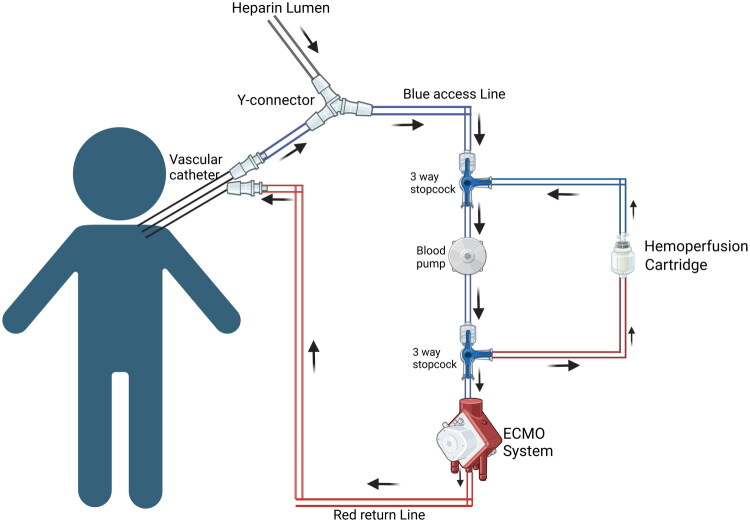
Hemoperfusion with ECMO. Circuit diagram showing integration of Hemadsorption with ECMO Created in BioRender. Hu, J. (2026) https://BioRender.com/rzvltqh

*Iwagami* et al. found no significant difference in mortality in patients with septic shock who received ECMO with PMX filter compared to ECMO alone [27]. A review by *Stegmayr* et al. found reduced 28-day mortality and notable improvements in hemodynamics and organ dysfunction in adult septic shock patients receiving only PMX adsorption therapy [28]. Another case report by *Kim* et al. showed significant short-lasting improvement in pulmonary oxygenation and hemodynamics in a neonate with septic shock, which led to weaning the patient from ECMO after tandem ECMO with a PMX filter [29]. However, pediatric mortality outcomes remain insufficiently studied, and no controlled trials have demonstrated a survival benefit in this population. Tables 2 & 3 describe a procedure to perform tandem hemoperfusion with CRRT and ECMO, respectively. Supplemental tables 1 & 2 describe the equipment required, monitoring, and completion of the procedure, as well as safety considerations. (Figure 3 and Figure 4)

**Table 2.** Procedure to integrate a hemoperfusion cartridge with CRRT.

Objective:
To safely integrate a Hemoperfusion cartridge into a continuous renal replacement therapy (CRRT) system for pediatric patients, utilizing a series connection with appropriate heparinization.
Procedure Steps:
*Preparation of CRRT System:*
Set up the CRRT system and ensure it is properly connected to the patient. Administer anticoagulation using heparin or another agent as per the standard CRRT protocol. Ensure all access lines (red access and blue return lines) are securely connected. Use a Y-connector to attach both the citrate lumen and the red lumen from the patient’s vascular catheter to this stopcock.
*Heparinization of Hemoperfusion Cartridge:*
Heparinize the Hemoperfusion cartridge by flushing it with a diluted heparin solution, ensuring all surfaces are coated, to prevent clot formation within the cartridge during therapy. Confirm that the cartridge is fully primed and no air bubbles remain in the system.
*Integration of Hemoperfusion Cartridge into CRRT Circuit:*
Proximal Red Access Line Connection: Add a three-way stopcock at the proximal site on the red access line of the CRRT device. Prime this stopcock and all associated tubing with normal saline. Connect the access line from the heparinized Hemoperfusion cartridge to the three-way stopcock, creating the proximal connection. Distal Red Access Line Connection: Add another three-way stopcock at the distal site on the red access line of the CRRT device. Prime the stopcock with normal saline. Connect the return line from the Hemoperfusion cartridge to this stopcock, completing the Hemoperfusion circuit. Blue Return Line Connection: Add a Y-connector to the blue return line from the CRRT device. Use this Y-connector to attach the calcium lumen and the blue lumen from the vascular catheter, completing the return flow pathway. Bypass Line: Maintain a bypass line between the two three-way stopcocks on the red access line to allow the CRRT system to function without the Hemoperfusion cartridge or during cartridge replacement. Ensure that the bypass line is primed and patent for quick access.

**Table 3.** Procedure to integrate a hemoperfusion cartridge with ECMO.

*Objective:*
To safely integrate a Hemoperfusion cartridge into an Extracorporeal Membrane Oxygenation (ECMO) system, using a series connection for optimal blood purification in pediatric patients.
*Procedure Steps:* *Preparation of ECMO System:*
Connect the ECMO device to the patient, ensuring that the circuit is securely established and anticoagulation is being administered via heparin as per ECMO protocol. Ensure all tubing and access lines (pre-pump and post-pump) are securely connected and primed with normal saline. Use a Y-connector to attach both the venous lumen from the patient and the heparin lumen to this stopcock.
*Heparinization of Hemoperfusion Cartridge:*
Heparinize the Hemoperfusion cartridge following the manufacturer’s instructions by flushing the cartridge with a diluted heparin solution. Ensure that the cartridge is fully primed with saline and that no air remains in the system before integration into the ECMO circuit.
*Integration of Hemoperfusion Cartridge into ECMO Circuit:* Pre-Pump, Pre-Oxygenator Connection: Add a three-way stopcock at the pre-pump, pre-oxygenator venous access line of the ECMO circuit. Prime this stopcock and its associated tubing with normal saline. Connect the return line from the Hemoperfusion cartridge to this stopcock. Post-Pump Venous Access Connection: Add another three-way stopcock at the post-pump venous access line of the ECMO circuit. Prime the stopcock and its associated tubing with normal saline. Connect the access line from the Hemoperfusion cartridge to this stopcock, completing the circuit.

## Tandem intermittent hemodialysis with hemoperfusion

Hemoperfusion (HP) is another type of EBP/ST, which is based on the absorbent and the target molecule interaction. It is hypothesized to be beneficial in COVID-19 patients with acute kidney injury (AKI) by removing cytokines and other inflammatory mediators from the blood [30–32]. In a retrospective cohort study by *Roberto* et al. eight patients with ESKD < 19 years of age presenting with moderate-to-severe COVID-19 underwent combined sessions of intermittent hemodialysis (IHD) and HP. Their serum levels of C-reactive protein, erythrocyte sedimentation rate, lactate dehydrogenase, and ferritin did not significantly change after 2–3 sessions; the decline in procalcitonin was significant after the second session (*p* = 0.046) [33]. Pneumonia on chest radiography progressively decreased across all sessions, with all patients showing clinical resolution of symptoms [33]. Additional research involving larger participant groups is required to investigate the correlation between the number of combined HP and HP sessions and changes in inflammatory markers. Although data in pediatric populations is limited, adults with diabetic ketoacidosis and acute renal failure demonstrated improvement in renal markers and symptoms on combined IHD and HP compared to HP alone [34].

### Tandem prolonged intermittent renal replacement therapy with hemoperfusion

Prolonged intermittent renal replacement therapy (PIRRT) can be defined as an EBP/ST method administered intermittently over a prolonged period, i.e. more than 6 h [35]. *Gong* et al. investigated the effects of combined PIRRT with HP in patients with moderate to severe pancreatitis and found a reduction in inflammatory markers, creatinine, and APACHE II score with the combined approach compared to controls who received only medical therapy [36]. A study by *Hernandez-Arago and Cruz* in 2023 compared the outcomes of PIRRT with or without HP in treating severe dengue among 18 patients aged ≤18 years [37]. Although the results from both approaches were similar, they reported that the combination of PIRRT with HP in hemodynamically unstable children with severe dengue was a feasible approach in resource-limited settings [37].In the future, more extensive studies are needed to evaluate the effectiveness of tandem PIRRT with HP in children, specifically concerning dialysis dosage and variations in cytokine levels or other inflammatory markers.

## Tandem continuous renal replacement therapy with leukapheresis

**Figure 5.**
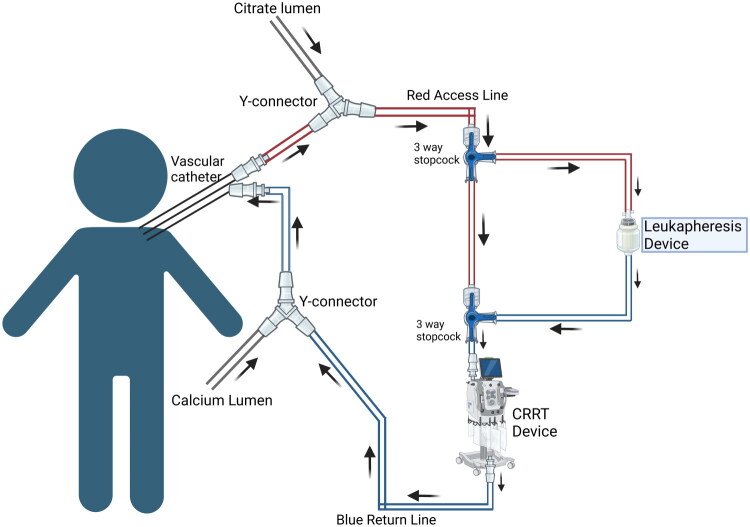
Leukopheresis with CRRT. Circuit diagram showing integration of leukopheresis with CRRT Created in BioRender. Hu, J. (2026) https://BioRender.com/ka4y0gg

Other extracorporeal blood purification therapies that could be combined with CRRT include leukapheresis. Leukapheresis is a nonsurgical therapy used in the initial management of leukostasis in a child with hyperleukocytosis, which reduces the concentration of white blood cells in the blood [38]. Leukapheresis was first used as a successful therapy by *Romano* et al. for leukodepletion in pediatric patients with pertussis in 2004, leading to subsequent oxygenation improvements [39]. Unlike other combinations of therapies, there is no published evidence in the literature about leukapheresis combined with CRRT in children. Perhaps both therapies are well established to treat critical illness in children. Rapid reduction of leukocyte burden in the setting of compromised renal function indicates the use of tandem leukapheresis with CRRT [38]. This combined approach through the same vascular access can be used to treat severe hyperleukocytosis with concurrent AKI or fluid overload in Tumor Lysis Syndrome, Multiorgan dysfunction, etc. Table 4 outlines the procedure to perform tandem Leukapheresis with CRRT in children (Figure 5). Supplemental Table 3 describes the equipment required, monitoring, and completion of the procedure, as well as safety considerations.

**Table 4.** Procedure to integrate a leukapheresis with CRRT.

*Objective:*
To safely perform a combined CRRT + leukapheresis procedure in pediatric or critically ill patients requiring both renal replacement and white blood cell depletion, ensuring optimal hemodynamic stability, anticoagulation balance, and circuit integrity. *Procedure Steps:*
Preparation Verify the correct setup of both CRRT and Spectra Optia^®^ systems. Confirm appropriate tubing sets, expiration dates, and patient-specific settings (weight, Hct, WBC count). Administer anticoagulation: Begin systemic heparinization as per CRRT protocol. For leukapheresis, ensure ACD-A infusion is connected to the Spectra Optia^®^ inlet line per manufacturer guidelines. Prime both systems individually using 0.9% NaCl until air-free. Integration of CRRT and Leukapheresis Circuits Proximal Access (Inlet Pathway): Add a three-way stopcock at the proximal red (access) line of the CRRT circuit. Connect the Spectra Optia^®^ inlet line (drawing blood for WBCD) to this stopcock. Ensure both CRRT and leukapheresis lines are primed with saline. Return Pathway (Effluent / Outflow): Add another three-way stopcock on the distal CRRT red line (pre-filter). Return the processed blood from Spectra Optia^®^ return line into this stopcock, maintaining laminar flow toward the CRRT filter. Use a Y-connector on the blue (return) line to merge the CRRT return with the patient’s venous return port. Bypass Line: Maintain a bypass loop between the two CRRT stopcocks for use during Spectra
Optia^®^ disconnection or alarm conditions. Operation Start CRRT and verify stable pressures (access < −150 mmHg, return < +200 mmHg). Activate Spectra Optia^®^ WBCD mode: Input patient data (height, weight, Hct, WBC count). Set target cell volume to process and desired depletion percentage. Select anticoagulant (ACD-A) and replacement fluid if indicated. Begin simultaneous operation: CRRT performs solute and fluid clearance. Spectra Optia^®^ performs selective leukocyte separation. Coordinate calcium infusion per Calcium SOP to prevent hypocalcemia during citrate infusion. Maintain continuous monitoring of inlet pressures, effluent flow, and anticoagulation rates.

Extra-corporeal supportive therapy like ECMO can also be used in tandem with Leukapheresis to treat children requiring cardio-vascular support along with rapid reduction of leukocytes. *Grzeszczak* et al. first reported using leukapheresis in combination with a patient already on ECMO support and demonstrated cardiovascular improvement [40]. Several studies since then have investigated this association between concurrent leukapheresis and ECMO initiation by adding a white blood cell filter to the ECMO circuit. Aggressive leukapheresis initiation with both ECMO and non-ECMO patients demonstrated elevated survival trends [6]. *Rowlands* et al. suggested initiating leukapheresis in patients with pertussis on ECMO with white blood cell counts greater than 50,000/μL [41].

Another study by *Domico* et al. looked at pediatric patients on ECMO for pertussis and found that higher mortality was associated with pulmonary hypertension, vasoactive infusion use, younger age, and a decreased PaO_2_/FiO_2_ ratio. Despite this, leukapheresis utilization demonstrated an independent, elevated chance of survival in this population [42]. *Scoble* et al. reported a case of AML with hypoxemia and leukocytosis refractory to ECMO with subsequent improvement in oxygenation following tandem ECMO and leukapheresis. However, the patient suffered hypoxic brain injury and expired [43].

**Figure 6.**
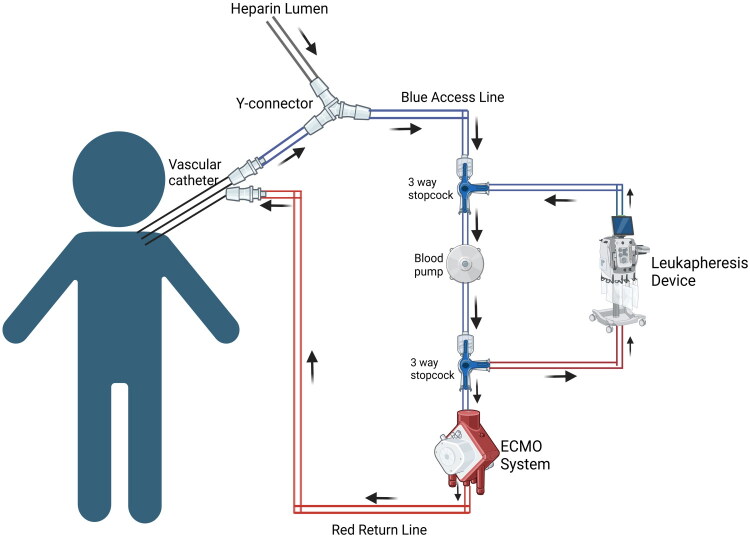
Leukopheresis with ECMO. Circuit diagram showing integration of leukopheresis with ECMO Created in BioRender. Hu, J. (2026) https://BioRender.com/n32t406

Leukapheresis systems such as Spectra Optia^®^ add an additional 160–185 mL of extracorporeal volume depending on configuration [44], which in small infants may exceed 30–40% of CBV. Notably, ultra-low extracorporeal volume microfluidic leukapheresis may represent a promising alternative to traditional high-volume centrifugation-based leukapheresis, although human studies are still required [45]. While recent literature has advocated for more restrictive use of leukapheresis in the setting of AML-associated hyperleukocytosis (ref below), it may still be considered on a case-by-case basis with shared decision-making with oncology teams, and providers should be aware of appropriate tandem therapy options with ECMO [46,47]. The protocol and prescription to administer leukapheresis with ECMO in children are explained in Table 5, and Figure 6 shows the circuit to integrate the leukapheresis device with ECMO. Supplemental Table 4 describes the equipment required, monitoring, and completion of the procedure, as well as safety considerations.

**Table 5.** Procedure to perform leukopheresis in tandem with ECMO.

Objective:
To safely perform a leukopheresis procedure in conjunction with an Extracorporeal Membrane Oxygenation (ECMO) system using the integration of both devices for efficient white blood cell depletion. Preparations:
Preparation of ECMO Circuit: Ensure the ECMO circuit is fully established and connected to the patient. Set up anticoagulation with heparin infusion as per ECMO protocol. Check all connections and ensure that the access lines (pre-pump and post-pump) are securely placed. Use a Y-connector to attach both the venous lumen from the patient and the heparin lumen into this stopcock. Integration of Leukapheresis into the ECMO Circuit: Pre-Pump Connection: Attach a three-way stopcock at the pre-pump, pre-oxygenator venous access line of the ECMO circuit. Prime the stopcock and its connections with normal saline. Connect the return line from the leukapheresis device to this same stopcock. 2. Post-Pump Connection: Attach another three-way stopcock at the post-pump venous access line. Prime the stopcock and its associated tubing with normal saline. Connect the access line from the leukapheresis device to this stopcock, completing the integration into the ECMO circuit. Priming and Setup of Leukapheresis System: Load and prime the leukapheresis tubing set using normal saline as per the leukapheresis device’s standard operating protocol. Ensure proper flow is established within the leukapheresis circuit. Patient Data Entry and Run Values: Enter patient-specific data into the leukapheresis system: Sex, height, weight, and hematocrit (Hct) to calculate total blood volume (TBV). White blood cell(WBC) count to set target values for depletion. Review and confirm the calculated TBV and WBC count, ensuring all parameters are set correctly. *Start of the Procedure:* Begin the leukapheresis procedure following the Spectra Optia operator manual. Ensure continuous heparin infusion is maintained as per the ECMO anticoagulation protocol. Monitor flow rates and pressure within the ECMO and leukapheresis circuits to ensure optimal blood exchange and WBC depletion. Adjust flow rates as needed to maintain patient stability and prevent hemodynamic complications.

## Other emerging blood purification therapies

Other emerging blood purification devices and filters are utilized for the removal of cytokines and endotoxins in patients undergoing extracorporeal support (Table 6). HA330 hemoperfusion adsorbent, made from Styrene divinylbenzene copolymers, is effective in adsorbing cytokines during KRT for septic shock and AKI [14]. Studies indicate that HA330 performs well in acute inflammatory conditions, particularly acute lung injury, with positive effects on CRP levels and heart rate, though it doesn’t significantly impact overall prognosis [48,49]. While the HA330 filter has shown a reduction in ICU mortality and improvement in oxygenation in cases like acute respiratory distress syndrome (ARDS), its effectiveness in COVID-19 pediatric patients remains uncertain [14]. Compared to continuous venovenous hemofiltration (CVVH) alone, combining hemoperfusion with CVVH enhances the removal of cytokines, potentially influencing outcomes in systemic and pulmonary inflammation [14,50]. Another emerging approach moves beyond cytokine adsorption toward direct modulation of the innate immune response.

**Table 6.** A summary of emerging blood purification devices.

*Device*	*Mechanism*	*Indication*	*Outcome*	*Adverse effects*
HA330^48^	Styrene divinylbenzene copolymer hemoperfusion adsorbent	Septic shock, AKI	Reduction in circulating cytokines.	Significant increase in BUN and decreased platelet count.
Selective Cytopheretic Device^49,50^	Biomimetic membrane	Septic Shock	Binding and sequestering of activated neutrophils.	No significant risk of device-related complications.
High cutoff (HCO) and medium cutoff (MCO) membranes^51,52^	HCO- dialysis membrane 8-10nm MCO - dialysis Membrane 3.5-5nm	AKI, septic shock	Reduction of: HCO- TNF-α, IL-6, and IL-10 MCO- high uremic toxins, β2-microglobulin, free light chains, myoglobin	HCO- Higher risk of albumin Loss MCO- β2-microglobulin, free light chains, myoglobin
Endotoxin removal-Toraymyxin (PMX)^53^	polymyxin B (PMX) bound to polypropylene-polystyrene fibers	AKI, Septic shock	Reduction of IL-6, IL8, and VEG. Conflicting evidence on removal of endotoxins.	Not statistically significant risk ratio of adverse events.
Polymethylmethacrylate (PMMA)^54^	PMMA	AKI	Reduction in IL-6, β2-microglobulin	No significant adverse events seen.

**Figure 7.**
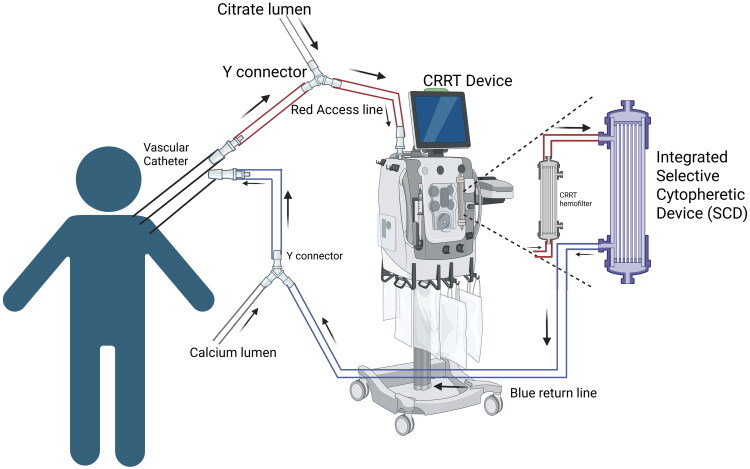
Tandem SCD with CRRT. Circuit diagram showing Tandem Selective Cytopheretic Device(SCD) with CRRT Created in BioRender. Hu, J. (2026) https://BioRender.com/ml9wg0t

The Selective Cytopheretic Device for pediatrics (SCD-PED) received FDA Humanitarian Device Exemption approval in February 2024. It is authorized for use in pediatric patients ≥10 kg with acute kidney injury due to sepsis or a sepsis-like condition who are receiving antibiotic therapy and require renal replacement therapy. This device operates by being connected in sequence with a CRRT system, employing regional citrate anticoagulation throughout the extracorporeal circuit. The SCD works through leukocyte immunomodulation in hyperinflammatory states, showing that in a pooled analysis of adult patients with AKI, SCD treatment led to a significant neutrophil-to-lymphocyte ratio reduction by day 6 (13.3 vs. 25.7; *p* = 0.011), maintained after sensitivity analysis (13.7 vs. 25.6; *p* = 0.013) [51]. In a case report by Goldstein et al. (2022), the use of an integrated SCD with CRRT facilitated a rapid recovery of both lung and kidney functions in a toddler diagnosed with hemophagocytic lymphohistiocytosis (HLH) [52]. In another case report, the SCD was used in conjunction with TPE and CRRT to treat a critically ill child with neutropenic sepsis with a PRISM III Score showing a 95% risk of mortality [53]. In a recent pediatric cohort study by Stanski et al. adjunctive use of SCD with CRRT (*n* = 18) was associated with shorter CRRT duration(median 6 vs 10 days, *p* = 0.013) and reduced ICU length of stay in survivors (16 vs 27 days, *p* = 0.012) compared with CRRT alone (*n* = 178) [54]. Survival to ICU discharge or day 60 was 94% vs 74%, though this did not reach conventional statistical significance (*p* = 0.079). The Bayesian analysis indicated >99% probability of improved survival, and in the sepsis subgroup, survival was 100% vs 69% (*p* = 0.032) with shorter CRRT duration (5 vs 11 days, *p* = 0.006) [54]. While Bayesian modeling suggested a high probability of survival advantage, these findings should be interpreted as hypothesis-generating rather than definitive evidence of mortality benefit. Another multicenter study of 22 pediatric patients receiving CRRT-SCD found that 77% survived to ICU discharge or day 60, compared with 55% in a matched historical CRRT cohort, with no SCD-related adverse events, indicating safety and probable benefit [55]. Humes et al. have shown that the SCD has been utilized in over 800 sessions and 19,000 clinical hours with no device-related infections or serious adverse events [56]. Although initial results have been promising, these findings derive from non-randomized comparisons and Bayesian modeling. The mechanistic rationale for SCD use derives from adult inflammatory models. Pediatric immune responses, particularly in neonates and infants, differ in neutrophil activation and cytokine regulation, which may influence device performance and clinical response. Further studies are required to validate its efficacy in the pediatric population, particularly in the context of COVID-19-associated AKI. Figure 7 shows the circuit to integrate the SCD with CRRT.

As immunomodulatory devices continue to evolve, membrane-based strategies remain a central component of extracorporeal support. High cutoff (HCO) and medium cutoff (MCO) membranes represent the next category of therapies aimed at enhancing cytokine clearance during CRRT. They are effective in filtering inflammatory molecules with molecular weights between 20 and 50 kDa like TNF-alpha and IL-6 [57,58]. When used with CRRT for cytokine removal, these membranes have demonstrated the ability to enhance oxygenation, alleviate non-cardiogenic pulmonary edema in critically ill patients and decrease ICU mortality [59].

Recently, United States FDA approved Toraymyxin, a Breakthrough Device designation for its treatment of patients with septic shock [60]. It consists of polymyxin B (PMX) bound to polypropylene-polystyrene fibers, primarily removing endotoxins extracorporeally rather than targeting cytokines [60,61]. A case series of COVID-19 patients with septic shock and AKI showed a 50% 28-day ICU mortality rate, with tandem PMX and CRRT use linked to hemodynamic recovery and improved organ function, but no PMX-related complications [60,61]. Katagiri et al. reported reduced inflammatory markers like IL-6, IL-8, and VEGF in adult patients treated with tandem PMX with hemoperfusion [62]. However, other larger studies found no significant reduction in mortality and endotoxin levels in the blood [63].

Although PMX primarily targets circulating endotoxin, additional strategies aim to reduce the inflammatory burden by adsorbing a wider spectrum of plasma proteins. The polymethyl methacrylate (PMMA) membrane represents one such modality with broader, nonspecific protein clearance capabilities. The polymethyl methacrylate (PMMA) membrane has ability to adsorb proteins such as IL-6, β2-microglobulin and it can remove high molecular weight proteins which cannot be efficiently cleared by CRRT alone [64]. *Miyamoto* et al. retrospectively studied PMMA in seven pediatric patients with AKI following cardiac surgery. Using PMMA with CRRT normalized blood urea nitrogen, lactate, and CRP levels, resulting in significant kidney improvement and no mortality [65].

**Figure 8.**
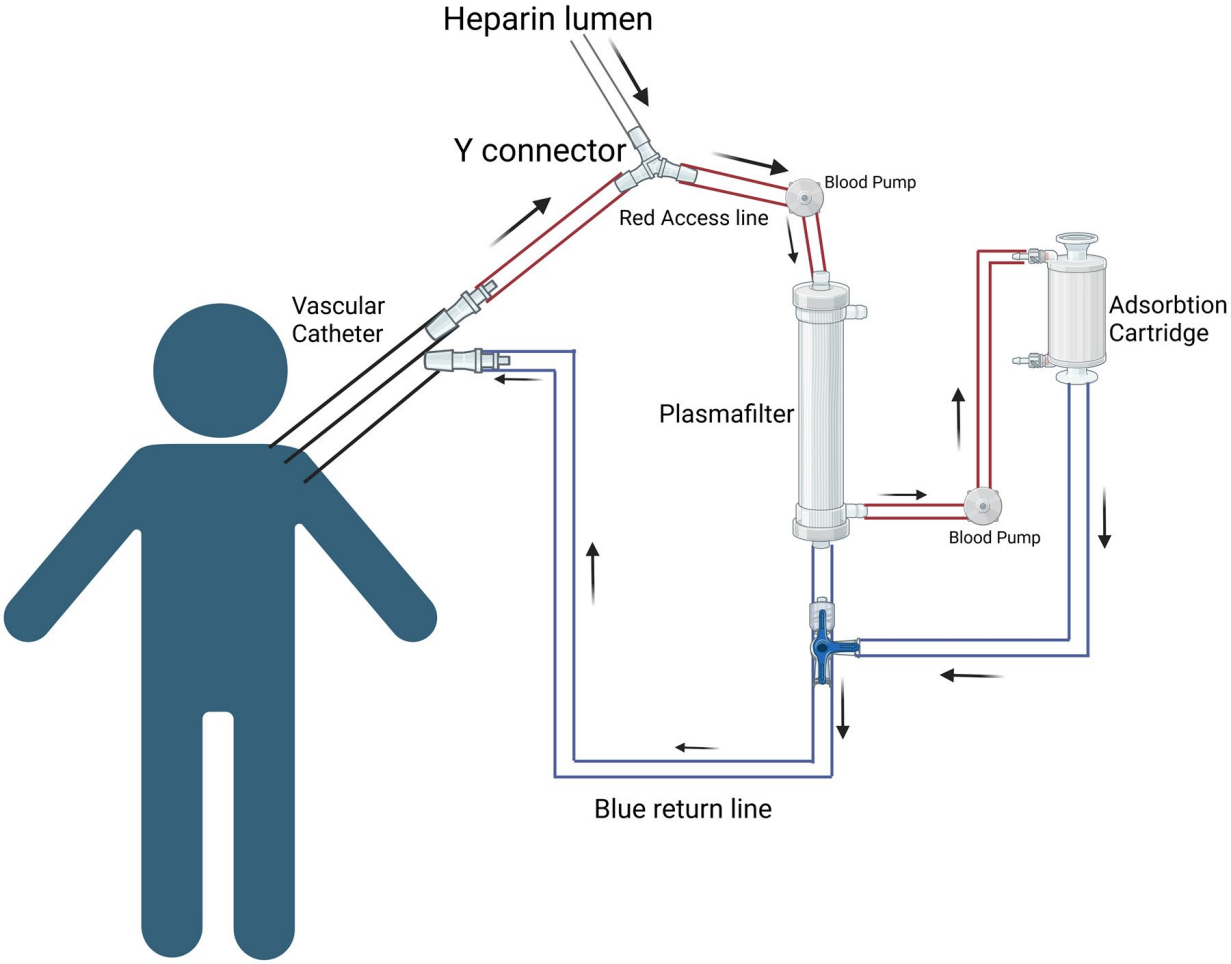
Tandem CPFA. Circuit diagram showing Tandem Coupled Plasma Filtration-Adsorption Created in BioRender. Hu, J. (2026) https://BioRender.com/83ezzdh

Coupled Plasma Filtration Adsorption (CPFA), another EBP/ST method, combines plasma filtration with an adsorbent cartridge and hemofiltration to eliminate cytokines and inflammatory mediators linked to severe sepsis and multiple organ dysfunction syndrome (MODS) [66,67]. A study by *Zhang* et al. has observed the rapid and significant decrease of plasma levels of liver-derived toxic substances like total bilirubin, direct bilirubin, and ammonia, and the improvement of coagulation dysfunction with prompt initiation of TPE combined with CPFA and chelation therapy in a 7-year-old presenting with severe hemolysis and impending Acute Liver Failure (ALF) [68]. Another study by *Hassan* et al. showed that treatment with CPFA + CVVH compared with CVVH alone in adult patients with severe sepsis resulted in earlier and sustained hemodynamic stability [69]. Tandem TPE plus CPFA is known to have a high detoxification capacity and allow efficient compensation of liver synthetic proteins, which may reduce the number of TPE sessions [70]. Table 7 has outlined the procedure to perform Coupled Plasma Filtration Adsorption (CPFA) in Children. (Figure 8)

**Table 7.** Procedure to perform coupled plasma filtration adsorption (CPFA) protocol in children.

Series Connection Setup: Use a series configuration for integrating Plasma Filtration-Adsorption with TPE or CRRT. Vascular Access Setup: Attach the red lumen of the vascular catheter and the heparin lumen to a Y-connector. Place the Y-connector at the proximal site on the access line of the circuit. Plasma Filtration and Adsorption Unit: Integrate the plasma filter on the main access line. Attach the adsorption cartridge’s access line to the distal outlet of the plasma filter. De-Aeration Chamber: Add a de-aeration chamber to the circuit after the plasma filter. Attach the return line from the adsorption cartridge to the de-aeration chamber. TPE/CRRT Integration: Integrate the TPE or CRRT device in series, placing it after the de-aeration chamber. Ensure the blood flows through the Plasma Filtration-Adsorption unit before entering the TPE/CRRT device. Return Line: Attach the return line from the TPE or CRRT device to the blue lumen of the vascular catheter, completing the circuit.

Lastly, Tandem Extracorporeal Liver Support (ELS) therapy refers to the artificial/bioartificial detoxification and synthetic replacement of liver functions in patients with Acute Liver Failure (ALF) or acute on chronic liver failure (ACLF) [71]. Some of the more utilized types include the Prometheus^®^ fractionated plasma separation and adsorption system, molecular adsorbent recirculating system (MARS^®^), and single-pass albumin dialysis (SPAD) [71].^78^A standard continuous renal replacement apparatus can be utilized for SPAD, involving a dialysate that contains albumin flowing in a counter-current direction of blood [72,73]. The MARS works similarly, with a countercurrent albumin mixture that then traverses a charcoal adsorber and an anion exchanger resin adsorber. The Prometheus device involves sending albumin-enriched plasma through resin and anion exchanger adsorber columns and then back to the primary circuit for water-soluble compound removal [71,74]. A case series by *Peek* et al. analyzed ALF and ACLF patients on MARS with veno-venous ECMO to treat elevated bilirubin levels. The study found ELS therapy improved survival in patients with ALF (RR = 0.70, p-value = 0.05), showing an overall 30% reduction in risk of death. However, ELS did not show a significant survival benefit in ALF patients [75]. Another study found a 64% survival rate in patients on ECMO + MARS, weaning off ECMO, compared to a 21% survival rate of weaning off ECMO alone [30]. Experience with tandem ELS during ECMO remains predominantly adult-based. Pediatric acute liver failure differs in etiology, regenerative capacity, and transplant candidacy, which may limit the direct applicability of adult outcome data.

## Anticoagulation considerations in tandem extracorporeal therapies

Anticoagulation management becomes substantially more complex in tandem extracorporeal configurations. CRRT commonly utilizes regional citrate anticoagulation (RCA) or systemic heparin, whereas ECMO typically requires continuous systemic heparinization, and leukapheresis employs citrate-based anticoagulants (ACD-A) [21]. When these systems are combined, cumulative anticoagulant exposure increases the risk of bleeding, citrate accumulation, metabolic alkalosis, and ionized hypocalcemia, particularly in neonates and patients with hepatic dysfunction. Concurrent systemic heparinization across ECMO and CRRT circuits may further predispose to bleeding complications and heparin-induced thrombocytopenia (HIT), while inadequate anticoagulation increases filter clotting and circuit loss. Adsorptive cartridges may also alter anticoagulant pharmacokinetics through nonspecific adsorption, potentially reducing effective heparin levels and contributing to unpredictable circuit performance [21]. In pediatric patients—who have limited blood volume reserves and evolving coagulation systems—the balance between hemorrhagic and thrombotic complications is especially delicate. Accordingly, tandem therapies require individualized anticoagulation protocols, frequent monitoring of activated clotting time (ACT), anti-Xa levels, ionized calcium, and circuit pressures to mitigate compounded risks.

## Emerging adsorptive and miniaturized technologies: translational implications for future tandem extracorporeal therapies

Although prior sections emphasize tandem extracorporeal blood purification strategies already implemented in pediatric clinical settings, recent developments in pre-clinical and translational research are uncovering mechanistic insights that may inform future configurations. Investigations of *in vitro* sorbent cartridge functionality, compact ultrafiltration platforms, and pharmacokinetic adsorption profiles offer foundational data for refining sorbent selection, optimizing circuit integration, and calibrating drug dosing in evolving extracorporeal support modalities. While these innovations are not yet incorporated into routine pediatric tandem therapies, their value lies in promoting evidence-based device pairing and anticipating potential iatrogenic effects.

Advances in sorbent-based technologies continue to influence the design of future tandem extracorporeal strategies for sepsis management. The usage of Cytokine Adsorption (CA) and Polymyxin B Hemoadsorption (pHA) cartridges for clearance of LPS endotoxin, IL-1β, and IL-6 cytokines offers a new therapy for the treatment of patients in septic shock from Gram-negative bacteremia. An *in vitro* simulation sought to provide insight into the potential use of CA and pHA cartridges for septic patients [76]. The adsorption capability of both cartridges was determined *via* an Endotoxin Activity Assay (EAA) level and the clearance of LPS, IL-1β and IL-6 were obtained through removal ratios (RR) over 120 min. Both CA and pHA cartridges significantly adsorbed LPS endotoxin, IL-1β and IL-6 cytokines [76]. The pHA cartridge reduced the EAA by 51.2% and the CA cartridge reduced the EAA by 65.9% at 120 min. Of note, the cartridges differed in their RR values. The IL-6 concentration of the CA cartridge had a RR of 49.2% within 10 min and 83.9% at 120 min while the pHA cartridge exhibited a more gradual reduction of IL-6 with an RR of ∼8% at 10 min and 49.2% at 120 min. IL-1β RR patterns were like the trends of IL-6 with the CA cartridge showing a more dramatic IL-1β clearance in the first 10 min when compared to the pHA cartridge with a final RR of 92.6% and 63.9% at 120 min respectively [76].

To broaden the therapeutic use of CA, the ability of five CA cartridges (XCA 1–5), each with a different prototype sorbent formulation were tested in an *in vitro* simulation of Gram-negative bacteremia [77]. Specifically, LPS endotoxin adsorption and IL-1β and IL-6 cytokine removal was compared over 120 min with LPS endotoxin adsorption capacity determined by EEA and LPS endotoxin and cytokine removal measured with removal ratios (RR). The CA sorbent formulation of XCA-2 and XCA-4 had the greatest adsorption of LPS endotoxin with an EAA of 83.3% and 81.1% respectively [77]. However, LPS endotoxin removal was found to be greatest in XCA-1 and XCA-5 with RR values of 13.2% and 13.3%, respectively. Regarding cytokine removal, XCA-3 showing the greatest RR for IL-6 at 80.9% and XCA-2 showing the greatest RR for IL-1β at 61.2% at 120 min. Overall, this study showed that there is not a single sorbent formulation that provides an all-encompassing, dominant clearance of LPS endotoxin, IL-1β and IL-6 cytokines [77]. Rather, sorbent concentrations should be tailored toward specific inflammatory markers. Although these findings originate from *in vitro* simulations, they hold relevance for tandem CRRT-based platforms by guiding the selection of sorbent cartridges aimed at predominant inflammatory mediators in the context of septic shock.

The path toward improving CRRT goes beyond the cartridges that are used, especially considering the potential for improving the accessibility, affordability and size of traditional RRT therapy. The AD-1 miniaturized ultrafiltration device may satisfy this need, offering a potential alternative to traditional RRT therapy [78]. Using the AD-1 lunchbox sized device, a comparison of physiologic filtration rates in adult male pigs and the artificial filtration rates of the AD-1 device was obtained [78]. A “target” filtration rate of 4.16, representing the ideal filtration rate in the average healthy adult male pig was matched *in vivo* with three healthy adult male pigs. The maximum average deviation from the target rate was no more than 10%. Ultimately, the AD-1 ultrafiltration device can simulate healthy filtration function in large animals indicating potential application in humans as a novel therapy for renal replacement [78].The advancement of miniaturized ultrafiltration systems, such as AD-1 platform, underscores a complementary trajectory of innovation focused on minimizing circuit complexity and extracorporeal volume—design attributes of particular importance for prospective pediatric tandem applications.

Beyond solute clearance, emerging evidence highlights the importance of understanding drug–membrane interactions within tandem extracorporeal circuits. Adsorptive membranes may also alter pharmacokinetics by nonspecifically removing circulating medications. Antibiotics such as linezolid and meropenem, certain immunosuppressants, and potentially vasoactive agents may be partially adsorbed during hemoperfusion or high-adsorption CRRT, leading to subtherapeutic exposure if dosing is not adjusted [79]. Although most available data derive from adult or *in vitro* studies, the risk may be amplified in pediatric patients due to smaller circulating blood volumes and variable drug distribution kinetics. The magnitude of drug removal depends on membrane characteristics, protein binding, molecular weight, and duration of therapy. Accordingly, incorporation of adsorptive cartridges should prompt consideration of therapeutic drug monitoring (when available) and dose adjustment strategies, particularly in critically ill children receiving time-sensitive antimicrobial or immunomodulatory therapy [79].

Collectively, these translational investigations emphasize that the future advances in tandem extracorporeal therapy will hinge not solely on circuit design, but also on the specificity of sorbent materials, the degree of device miniaturization, and the integration of pharmacokinetic principles—all of which are critical to ensuring the safe and effective expansion of tandem EBP/ST in pediatric critical care settings.

## Evidence classification framework

Given the heterogeneity of available data, evidence cited in this review is categorized using a simplified framework to differentiate between levels of support: Level A – Pediatric Controlled Data: Randomized or prospective pediatric trials; Level B – Pediatric Observational Data: Pediatric cohort studies, case series, or registry analyses; Level C – Adult Clinical Data: Randomized or observational adult studies extrapolated to pediatric contexts; Level D – Preclinical or Translational Data: *In vitro* studies, animal models, or mechanistic simulations.

This framework is intended to provide rapid contextualization of evidentiary strength and to minimize inadvertent overinterpretation of adult or mechanistic findings as validated pediatric efficacy. Using this evidence framework, most tandem extracorporeal therapies in children currently fall within Level B (observational pediatric data) or Level C (adult extrapolation), with several emerging platforms supported only by Level D translational data. Advancement to Level A pediatric-controlled evidence remains a critical research priority.

## Barriers to implementation

In tandem with extracorporeal therapies, domain-specific barriers compound these system issues. These platforms are largely adult-oriented; high priming volumes, circuit dead space, and connector mismatches complicate their use in children. Regulatory pathways are unclear, leading to the use of off-label configurations without explicit support for integrated multi-device operation. Combining CRRT, ECMO, adsorption cartridges, and/or leukapheresis increases circuit complexity, vascular access manipulation, and monitoring demands. This layered integration may elevate the risk of access-related complications, circuit misconnection, alarm fatigue, workflow interruptions, and human error. As device numbers and circuit interfaces increase, so does the need for protocol standardization, simulation-based team training, and clearly defined troubleshooting pathways to ensure safe implementation. Also, costs for cartridges, disposables, and parallel staffing are high, while coding rarely captures true resource use; the lack of standardized protocols and limited cross-trained staff further hinders reliable execution. Interoperability gaps with EMRs impede quality improvement and research. Also, supply-chain fragility (cartridge availability, shelf-life, service support) adds operational uncertainty.

Age- and weight-specific vulnerabilities further amplify risks in neonatal and infant populations. Neonates have limited thermoregulatory capacity, and exposure to extracorporeal circuits increases the risk of hypothermia, particularly during circuit priming and initiation. Immature renal and hepatic function predisposes to more pronounced electrolyte shifts, including rapid sodium, potassium, and calcium fluctuations during CRRT or citrate anticoagulation [21]. Additionally, smaller circulating blood volumes increase susceptibility to circuit-related anemia from hemodilution, repeated laboratory sampling, and blood loss during filter clotting or circuit changes [21]. These physiologic constraints render neonates and small infants disproportionately vulnerable compared with older children and necessitate weight-adjusted circuit design, temperature control strategies, and careful electrolyte and hemoglobin monitoring.

As identified by New York State Medicaid, barriers to implementing tandem therapy largely stem from complex billing procedures. The New York State Collaborative Care Program is an evidence-based approach that integrates mental health services into primary care settings [75]. Through surveying, the program recorded that clinics experienced issues with navigating billing codes and reimbursement. When clinics implemented tandem therapy in their care, the reimbursement rates of the clinics decreased, as a mismatch occurred between the amount that the clinic could bill and the reimbursement the clinic received. In addition, many clinics lacked dedicated staff to manage billing and data reporting, increasing the burden on administration [80]. Limited buy-in of the Collaborative Care model from organizations and providers who may not fully understand tandem therapy hampers the implementation efforts. Finally, varying rules about who can provide behavioral health services restrict the flexibility of clinics to deliver tandem therapy effectively [80].

## Conclusion

Tandem EBP/ST modalities beyond TPE, CRRT, and ECMO appear technically feasible and biologically plausible in critically ill children. However, current evidence is insufficient to confirm improvement in survival or other patient-centered outcomes, and most reported benefits remain limited to surrogate biomarker or physiologic endpoints. Available data are derived largely from small studies with variable patient selection, device usage protocols, and non-standardized outcome reporting. Coordinated research efforts are needed to better define the role of these tandem modalities in critically ill children. While adult data provide important mechanistic insight, pediatric-specific evidence remains limited, and careful interpretation is required to avoid direct extrapolation. Adequately powered pediatric studies are needed to establish safety, efficacy, and optimal patient selection criteria.

Advancing these therapies toward standardized pediatric care will require prospective multicenter registries, pragmatic or randomized trials, and consensus guidance addressing timing, device combinations, dosing intensity, anticoagulation, monitoring, and de-escalation. Future studies should evaluate clinically meaningful endpoints beyond short-term survival, including organ recovery, neurodevelopmental outcomes, and long-term kidney health. Parallel translational work should refine biomarker-guided personalization, characterize drug–device interactions within adsorptive circuits, and validate miniaturized, low-extracorporeal-volume platforms suitable for neonates and small children. Future implementation efforts should also address interoperable electronic health record–based data capture, pediatric-specific hardware, regulatory pathways for integrated multi-device use, cost-effectiveness, and workforce training to support equitable and reproducible practice across centers.

## Supplementary Material

Final Supplemenatary Doc Tandem therapy.docx

## Data Availability

Data sharing does not apply to this article as no new data were created or analyzed in this research.
