# Supplementary material for: Tandem extracorporeal blood purification therapies for sepsis and acute kidney injury in critically ill children: a state-of-the-art review
Source: Ren Fail. 2026 Apr 14;48(1):2653939. doi: 10.1080/0886022X.2026.2653939 (PMC13081349; doi:10.1080/0886022X.2026.2653939)
Supplement: Final Supplemenatary Doc Tandem therapy.docx [file IRNF_A_2653939_SM4169.docx]

**­­­­Title: Tandem Extracorporeal Blood Purification Therapies for Sepsis and Acute Kidney Injury in Critically Ill Children: A State-of-the-Art Review**

**Corresponding author:**

Rupesh Raina, MD, FAAP, FACP, FASN, FNKF.

Department of Nephrology, Akron Children’s Hospital,

214 West Bowery Street, Akron, OH 44308, USA;

Department of Nephrology, Akron Nephrology Associates/Cleveland Clinic Akron General Medical Center, Akron, OH, USA.

Email ID: [rraina@akronchildrens.org](mailto:rraina@akronchildrens.org)

**Running Headline**: Tandem therapies in critically ill children

**Key Words:** Pediatric critical care; Acute kidney injury; Sepsis; Extracorporeal blood purification; Continuous renal replacement therapy; Extracorporeal membrane oxygenation

| *Objective:* To safely integrate a Hemoperfusion cartridge into a continuous renal replacement therapy (CRRT) system for pediatric patients, utilizing a series connection with appropriate heparinization. *Equipment and Supplies:*  1. CRRT System: CRRT machine and tubing; Heparinized saline or other anticoagulation solution as per CRRT protocol; Y-connectors; Three-way stopcocks (multiple) 2. Hemoperfusion Cartridge: Hemadsorption cartridge; 0.9% Sodium Chloride (Normal Saline) for priming; Heparin for heparinization of the Hemoperfusion cartridge as needed (based on manufacturer’s recommendations)   *Monitoring During the Procedure:*   1. Closely monitor the patient for any signs of complications such as Hemodynamic instability, Electrolyte imbalances (e.g., citrate or calcium). 2. Regularly assess laboratory values including electrolyte levels, blood gas analysis, and clearance levels for targeted substances. 3. Adjust settings as required to maintain optimal function of both the Hemoperfusion cartridge and CRRT system.   *Cartridge Replacement or Bypass:*   1. If the Hemoperfusion cartridge needs to be replaced, switch to the bypass line temporarily to maintain CRRT function while replacing the cartridge. 2. Ensure sterile transitions during cartridge replacement to prevent interruptions in treatment and minimize infection risk.   *Completion of the Procedure:*   1. Once Hemoperfusion therapy is completed, discontinue the Hemoperfusion cartridge. 2. Disconnect the Hemoperfusion cartridge following standard post-apheresis or post-Hemoperfusion care protocols. 3. Continue CRRT therapy based on the patient's needs. 4. Monitor the patient for delayed complications such as electrolyte imbalances and hemodynamic instability. 5. Document all procedural details, including any interventions or adjustments during treatment.   *Safety Considerations:*   - Ensure that the heparinization of the Hemoperfusion cartridge is properly executed to prevent clotting. - Maintain consistent anticoagulation across the CRRT and Hemoperfusion circuits to avoid clot formation. - Monitor flow rates and patient tolerance to prevent hemodynamic instability. |
| --- |

**Supplemental Table 1:** Procedure to integrate a Hemoperfusion cartridge with CRRT.

**Supplemental Table 2:** Procedure to integrate a Hemoperfusion cartridge with ECMO

| *Objective:*  To safely integrate a Hemoperfusion cartridge into an Extracorporeal Membrane Oxygenation (ECMO) system, using a series connection for optimal blood purification in pediatric patients.  *Equipment and Supplies:*   1. ECMO System: ECMO machine and tubing; Heparinized saline or other anticoagulation solution as per ECMO protocol; Y-connectors; Two three-way stopcocks 2. Hemoperfusion Cartridge: Hemoperfusion cartridge; 0.9% Sodium Chloride (Normal Saline) for priming; Heparin for heparinization of the cartridge   *Monitoring During the Procedure:*   1. Monitor the patient for signs of complications such as Hemodynamic instability, Electrolyte imbalances. 2. Regularly assess laboratory values, including blood gas analysis and coagulation parameters, to ensure the therapy is running smoothly.   *Cartridge Replacement or Bypass:*   1. If the Hemoperfusion cartridge needs to be replaced during therapy, temporarily disconnect the cartridge and continue ECMO support using a bypass line. 2. Ensure sterile transitions during cartridge replacement to prevent contamination or interruptions in treatment.   *Completion of the Procedure:*   1. Once the Hemoperfusion treatment is complete or the patient’s therapeutic goals are achieved, disconnect the Hemoperfusion cartridge from the ECMO circuit. 2. Continue ECMO therapy as per the patient’s condition or treatment plan. 3. Ensure proper documentation of the procedure, including any changes in settings or complications that arose during treatment. 4. Document all procedural details and any complications or interventions during the process.   *Safety Considerations:*   - Heparinization of the Hemoperfusion cartridge is crucial to prevent clotting during therapy. - Continuously monitor flow rates and patient hemodynamics to prevent clot formation or hemodynamic instability. - Ensure that the ECMO and Hemoperfusion circuits function in synergy without interfering with the patient’s overall treatment stability. |
| --- |

**Supplemental** **Table 3:** Procedure to integrate a leukapheresis with CRRT:

| Objective: To safely perform a combined CRRT + leukapheresis procedure in pediatric or critically ill patients requiring both renal replacement and white blood cell depletion, ensuring optimal hemodynamic stability, anticoagulation balance, and circuit integrity.  Equipment and Supplies   1. CRRT System:    - CRRT machine (e.g., Prismaflex, Aquarius, or NxStage)    - Standard CRRT tubing set and dialysate/replacement fluids    - Y-connectors and multiple three-way stopcocks    - Heparinized saline or other anticoagulation per institutional protocol 2. Leukapheresis System:    - Spectra Optia® Apheresis System (WBCD mode)    - Spectra Optia® IDL set with filler    - 0.9% Normal Saline for priming    - ACD-A anticoagulant (Citrate Dextrose Solution A)    - Calcium chloride (8 mg/mL) or calcium gluconate (20 mg/mL) in 0.9% NaCl for citrate-induced hypocalcemia    - Replacement fluid if prescribed (e.g., plasma or albumin)    - Blood warmer and warmer tubing (if required) 3. Vascular Access:    - Dual-lumen central venous catheter with adequate flow rates (≥ 6 Fr in pediatrics)    - Peripheral venous access may be used for leukapheresis return line if feasible   4. Monitoring During Combined Therapy   - Observe for hemodynamic instability, filter clotting, and citrate toxicity (perioral tingling, hypotension). - Perform laboratory monitoring: ionized calcium, electrolytes, ABG, and WBC count at regular intervals. - Adjust anticoagulant (heparin/ACD-A) and calcium infusion rates to maintain circuit patency and patient safety. - Ensure flow rates of both circuits remain within manufacturer-recommended limits to avoid negative pressure alarms.   5. Cartridge / Tubing Maintenance   - In case of leukapheresis set alarm or completion:   - Pause both systems, clamp lines, and activate CRRT bypass mode.   - Disconnect Spectra Optia® per sterile technique; replace or dispose of tubing set.   - Resume CRRT alone if indicated.   6. Completion of the Procedure   1. Stop Spectra Optia® run upon reaching target depletion or physician order. 2. Perform rinseback per Spectra Optia® protocol to return blood in the extracorporeal circuit to the patient. 3. Continue CRRT independently for renal support as required. 4. Record all parameters, processed volumes, anticoagulation rates, calcium supplementation, and observed complications.   Safety Considerations   - Ensure all stopcocks and connectors are tightly secured to prevent air embolism. - Maintain consistent anticoagulation between circuits—avoid heparin + citrate imbalance. - Prevent excessive negative pressures by synchronizing pump flow rates. - Monitor for signs of filter clotting, hypocalcemia, or hypotension throughout the procedure. - Use blood warmer if required to maintain normothermia. - Document the entire procedure per institutional protocol and manufacturer guidance. |
| --- |

**Supplemental Table 4:** Procedure to perform leukopheresis in tandem with ECMO.

| *Objective:* To safely perform a leukopheresis procedure in conjunction with an Extracorporeal Membrane Oxygenation (ECMO) system using the integration of both devices for efficient white blood cell depletion. *Equipment and Supplies:*  1. ECMO Circuit: ECMO machine and tubing; Heparinized saline for anticoagulation; Y-connectors; Two three-way stopcocks 2. Leukapheresis Setup: Leukapheresis device (Spectra Optia® preferred); Leukapheresis tubing set; 0.9% Sodium Chloride (Normal Saline) for priming; Blood warmer and warmer tubing (if required)  *Start of the Procedure:*  1. Begin the leukapheresis procedure following the Spectra Optia operator manual. 2. Ensure continuous heparin infusion is maintained as per the ECMO anticoagulation protocol. 3. Monitor flow rates and pressure within the ECMO and leukapheresis circuits to ensure optimal blood exchange and WBC depletion. 4. Adjust flow rates as needed to maintain patient stability and prevent hemodynamic complications.  *Monitoring During the Procedure:* Monitor the patient for any complications, such as hemodynamic instability, electrolyte imbalances  1. Regularly assess laboratory values, including blood gas analysis and WBC count, and adjust treatment as necessary.  *Completion of the Procedure:*  1. Once the target WBC depletion is achieved, slowly wean the patient off the leukapheresis system. 2. Disconnect the leukapheresis device following standard post-procedure protocols. 3. Continue ECMO therapy based on the patient's treatment plan.  *Post-Procedure Care:*  1. Continue to monitor the patient for any delayed complications such as bleeding or hemodynamic instability. 2. Evaluate post-apheresis WBC counts and adjust the treatment plan as necessary. 3. Document all procedural details, including any complications or interventions during the process.  *Safety Considerations:*  - Ensure anticoagulation with heparin is properly managed between ECMO and leukapheresis circuits to prevent clotting or excessive bleeding. - Continuously monitor flow rates and patient tolerance to prevent hemodynamic instability. |
| --- |
